# Complex regional pain syndrome–up-to-date

**DOI:** 10.1097/PR9.0000000000000624

**Published:** 2017-10-05

**Authors:** Frank Birklein, Violeta Dimova

**Affiliations:** Department of Neurology, University Medical Centre of the Johannes Gutenberg University Mainz, Mainz, Germany

**Keywords:** Complex regional pain syndrome, Posttraumatic inflammation, Neuroplasticity, Central reorganisation, Treatment

## Abstract

Complex regional pain syndrome (CRPS) was described for the first time in the 19th century by Silas Weir Mitchell. After the exclusion of other causes, CRPS is characterised by a typical clinical constellation of pain, sensory, autonomic, motor, or trophic symptoms which can no longer be explained by the initial trauma. These symptoms spread distally and are not limited to innervation territories. If CRPS is not improved in the acute phase and becomes chronic, the visible symptoms change throughout because of the changing pathophysiology; the pain, however, remains. The diagnosis is primarily clinical, although in complex cases further technical examination mainly for exclusion of alternative diagnoses is warranted. In the initial phase, the pathophysiology is dominated by a posttraumatic inflammatory reaction by the activation of the innate and adaptive immune system. In particular, without adequate treatment, central nociceptive sensitization, reorganisation, and implicit learning processes develop, whereas the inflammation moderates. The main symptoms then include movement disorders, alternating skin temperature, sensory loss, hyperalgesia, and body perception disturbances. Psychological factors such as posttraumatic stress or pain-related fear may impact the course and the treatability of CRPS. The treatment should be ideally adjusted to the pathophysiology. Pharmacological treatment maybe particularly effective in acute stages and includes steroids, bisphosphonates, and dimethylsulfoxide cream. Common anti-neuropathic pain drugs can be recommended empirically. Intravenous long-term ketamine administration has shown efficacy in randomised controlled trials, but its repeated application is demanding and has side effects. Important components of the treatment include physio- and occupational therapy including behavioural therapy (eg, graded exposure in vivo and graded motor imaging). If psychosocial comorbidities exist, patients should be appropriately treated and supported. Invasive methods should only be used in specialised centres and in carefully evaluated cases. Considering these fundamentals, CRPS often remains a chronic pain disorder but the devastating cases should become rare.

## Abstract 2

The pathophysiology of complex regional pain syndromes includes inflammation and central reorganisation. The treatment should be adjusted to the prevailing pathophysiology including possible psychosocial factors.

**Key Points**

The pathophysiology of complex regional pain syndrome has become clearer through research in recent years. The pathophysiology translates into clinical symptoms, which can be identified. Treatment should be individually tailored according to the predominant pathophysiology. This is outlined in this article.

## 1. The history of complex regional pain syndrome

It took approximately 100 years to form the acronym “CRPS.” In 1864, Silas Weir Mitchell reported on patients whose disease corresponds to what we now call complex regional pain syndrome (CRPS) type II (Causalgia).^61^ In 1901, Paul Sudeck from Hamburg, Germany, described the “acute reflex bone atrophy after inflammation and injuries of the extremities and their clinical appearances,” which corresponds to CRPS type I without nerve lesion.^87^ The next milestone in CRPS history was reached in 1936, when James A. Evans coined the phrase “reflex sympathetic dystrophy”, which has been used for decades.^31^ At a conference in Orlando, 1995, it was agreed to use the descriptive phrase “Complex Regional Pain Syndrome” to avoid claims about pathophysiology.^86^

## 2. Principal factors for development and prognosis

Complex regional pain syndrome usually develops after an injury of the extremities. The latency between the injury and the earliest CRPS diagnosis depends on the “normal” time of recovery from injury. For an uncomplicated radial fracture, a recovery of 4 to 6 weeks is typically realistic. Complicated injuries take longer to recover. Thereafter, a diagnosis of CRPS could be made (point 1 of the diagnostic criteria; see below). Women aged between 40 and 60 seem to be most frequently affected. The female preponderance, however, could also be an artefact because women suffer 3 times more radial fractures than men.^45^

The risk of CRPS seems to be higher for patients with complicated fractures, a rheumatological disease, or intense pain (>5 on a 11-point numerical rating scale) 1 week after trauma.^65,84^ Epidemiological data from 2 major studies show a CRPS incidence between 5.5^77^ and 26.2 cases^25^ per 100,000 people per year. The variation may result from the use of different diagnostic criteria. It is only in the last decade that the validated “Budapest Criteria” (see below) have become generally accepted.

Regarding the prognosis, Bean et al reported in a longitudinal study that within the first year, 70% improved, especially in the function of the extremity and the visible symptoms (edema, skin color, and sweating). However, 25% of the patients still fulfilled the Budapest Criteria and only 5% were without complaints.^5,8^ Patients reporting higher levels of anxiety and pain-related fear at the beginning of therapy have worse long-term outcomes after 1 year.^6^

## 3. Classification and diagnosis

The diagnosis of CRPS is made clinically using the diagnostic criteria of the “IASP”^38^ (Table 1). It can be differentiated between CRPS type I, without obvious nerve lesion and CRPS type II, with verifiable nerve lesion. At first presentation, approximately 70% of patients report about a “primarily warm” subtype with an increased skin temperature at symptom onset, whereas the remaining 30% report a “primarily cold” subtype.^13^ Typically, a trauma precedes the clinical symptoms; “spontaneous” CRPS is rare and needs an extensive clarification of differential diagnoses because it is important to notice point 4 of the diagnostic criteria: “There is no other diagnosis that better explains the symptoms.” Unfortunately, the fact that CRPS usually affects distal limbs (an exception might be the knee) is not mentioned and neither is the fact that the signs must go beyond single-nerve innervation territories. Despite case reports, the authors doubt that CRPS of large joints, face, or trunk exists.

**Table 1:**
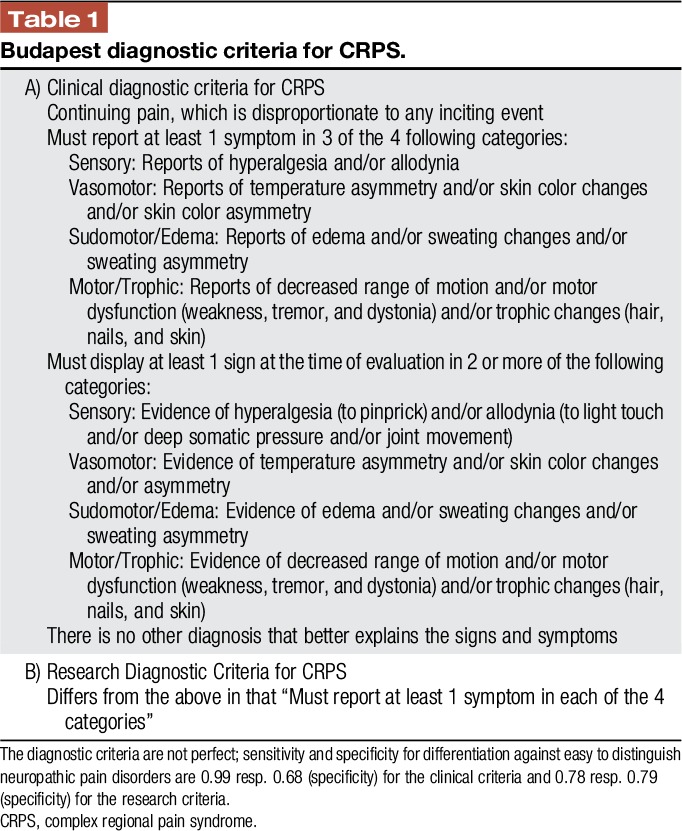
Budapest diagnostic criteria for CRPS.

Instrument-based investigations might be beneficial if there are doubts concerning the differential diagnoses. (1) Repeated measurements of the skin temperature show dynamics, ie, changing temperature differences (warmer gets colder or vice versa) of >1°C^51^; (2) Limb magnetic resonance imaging helps to exclude differential diagnoses like rheumatic diseases or infections; (3) x-rays in direct side-to-side comparison are not sensitive but can prove a patchy osteoporosis or may help to make differential diagnoses such as a pseudoarthrosis after fracture; (4) the 3-phase bone technetium scintigraphy in acute (but not chronic) CRPS has a 70% specificity and sensitivity compared to the clinical diagnostic criteria if there is evidence of an increased bone metabolism, typically in distal joints.^101^

Quantitative sensory testing (QST), which has become important in academic pain medicine, is not suited to make a CRPS diagnosis because QST generally describes pain symptoms (eg, hyperalgesia), which are not specific for any pain disorder. However, a typical QST pattern (thermhypaesthesia, mechanical hyperalgesia, and pressure hyperalgesia) may support a CRPS diagnosis, particularly if the distal joints, which were not directly affected by the trauma, are sensible to pressure pain.^57^

The CRPS severity score (Table 2)^39^ might be an instrument to grade the severity of CRPS and helps to monitor the course. Very low scores support considering a differential diagnosis (Table 2).

**Table 2:**
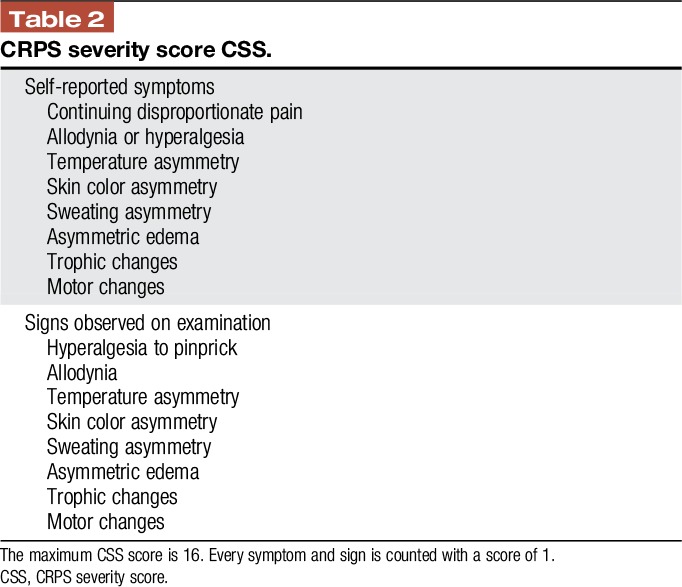
CRPS severity score CSS.

## 4. Clinical symptoms

Pain is the most important symptom. It is permanent or fluctuating and most often in the deep tissue. It increases through movements and during changes in temperature; in the experience of the authors, especially in chronic and severe cases, allodynia is a hallmark.^12^ At the same time, sensory deficits are reported: hypoesthesia and impairment of thermal perception^17^ after a glove- or stocking-like pattern. Patients report feelings that their extremity no longer belongs to their body.^33^ All patients have decreased muscle strength and probably pain-induced movement avoidance.^12^ Contractures develop quickly. Although the decrease in strength and the inhibition of movement both improve with reduction in pain, contractures improve slowly and sometimes remain permanent. Mainly poorly treated acute (<3–6 months from the onset) CRPS cases might develop shortening and fibrosis of capsules and tendons.^100^ In some cases, this could happen regardless of any treatment attempt. Other trophic changes can be found on the skin (eg, ulcers), the nails, and the hairs (in acute CRPS increased and in chronic CRPS decreased growth).^58^ The main symptom of vascular dysfunction is the oedema which can grow to dramatic extents and is always found in the acute phase.^12^ In chronic CRPS, patients report their own extremity often thicker than it actually is.^72^ Fifty percent of the patients have sudomotor disturbances, mostly a hyperhidrosis.^12^ All patients display a change in skin colour from reddish (“warm” CRPS) to blueish livid (“cold” CRPS).^11,13^ Skin temperature is different when comparing both sides.^94^ More rare are tremor, myoclonus, or fixed dystonia.^93^

## 5. Pathophysiology of complex regional pain syndrome

### 5.1. Exaggerated inflammation

It is heavily discussed whether there is a genetic disposition for CRPS. There are “CRPS families”^42^ and striking associations to migraine.^74^ Associations with known gene polymorphisms have been described in smaller studies but could not be replicated in larger cohorts.^44^ As long as we do not have biomarkers for subgrouping, the detection of genetic factors will remain difficult. Furthermore, association studies need high numbers, but CRPS is a rare disease. MicroRNAs are “master switches” for complex inflammatory reactions and pain states because they control the translational process for several proteins at the same time. If replicated, the pattern of microRNAs in plasma exosomes for cell–cell communication might be useful to identify patients with CRPS early after a trauma.^59^

The first step of CRPS pathophysiology is posttraumatic inflammation, mainly in “warm” CRPS, during the acute phase of the disease. Clinical observation finds signs of inflammation like redness, swelling, hyperthermia, pain, and reduced function^58^ (c.f. Galen, ∼210 A.D.). A trauma causes a complex immune response. In the skin, keratinocytes proliferate and produce inflammatory cytokines as part of the innate immune system.^10^ The cytokines (from keratinocytes, endothelial or immune cells) themselves proliferate connective tissue cells leading to contractures.^9^ Cytokines activate osteoblasts and osteoclasts, which explains the osteoporosis.^98^ Cytokines provoke pain and hyperalgesia through sensitization of peripheral nociceptors, and they facilitate the release of neuropeptides from nociceptors,^69^ which in turn are responsible for the “visible” inflammatory signs. Calcitonin gene–related peptide and substance P are released from the cytokine-sensitized nociceptors (neurogenic inflammation) and cause reddening, warmth, and edema^36,96^; substance P further promotes hair growth,^71^ and calcitonin gene–related peptide enhances sweating.^80^ Throughout the course of CRPS, most of these signs normalize, which demonstrates some change in pathophysiology.^54^ Recent investigations suggest a contribution of the adaptive immune system as well. The detection of agonistic serum auto-antibodies against adrenergic and cholinergic receptors renders an auto-immune component of CRPS very likely.^30,50^ Inflammation is less obvious in primarily cold CRPS, and investigations specifically for this subtype are sparse. Increased endothelin 1 and reduced nitric oxide probably contribute to the cold bluish skin.^36^ In preclinical studies, we found that a lack of neutral endopeptidase (reduced activity of peptidases is 1 hypothesis for a susceptibility for CRPS after trauma^26^) increases endothelin-1, which in turn sensitizes C-fibres.^41^

### 5.2. Central reorganization

The next step in CRPS pathophysiology is neuronal plasticity in the CNS, which is either induced by inflammation or develops in parallel. Plasticity is important, especially for CRPS, which is treatment resistant for more than 6 to 12 months, when symptoms cannot be explained through peripheral pathophysiology alone. In part, those symptoms can be attributed to learning processes, ie, “learned non-use” because of movement-related pain avoidance.^75^ Another possibility is a reflex inhibition of movement mediated by the expectation of pain.^89^ This results in a pathological movement pattern (eg, while walking), which again increases the pain through eg, unphysiological muscle and joint loads.^27,55^ Other symptoms are a direct consequence of reorganisation of somatosensory function in the brain^22,53^: body midline is shifted towards the healthy side and the CRPS extremity is perceived as distorted.^64^ The perception of allodynia is a consequence of central (spinal) sensitization. Its presence has been verified through functional magnetic resonance imaging by activation of the “pain matrix” through painful touching of the affected but not by nonpainful touching of the unaffected hand.^56^ For details on functional imaging in CRPS, we refer to an upcoming review.^88^

### 5.3. Reflex? sympathetic? dystrophy?

The significance of a sympathetic nervous system dysfunction for CRPS development has been questioned. Many of the presumably sympathetic symptoms like edema, vasodilatation, or hyperhidrosis can be explained through inflammation.^80,97^ However, inflammatory processes fade within the first year. If visible autonomic symptoms (eg, cold bluish skin, edema, and sweating changes) remain, they must have another pathophysiology, eg, sympathetic dysfunction as a consequence of central reorganisation.^34^ If patients with chronic CRPS think of a movement which would be painful, they activate the sympathetic nervous system.^66^ The skin temperature minimally changes when crossing over the hands bringing the CRPS hand into the healthy “peripersonal” space.^64^

In addition, peripheral adrenoreceptors of the affected tissue develop supersensitivity^2^ supposedly through the inflammatory processes within the first months. This supersensitivity causes activation of the sympathetic nervous system, which is normally symmetrical, leads to asymmetrical sympathetic symptoms. The hypothesis of “sympathetically maintained pain” is similar: nociceptors in the affected limb become sensitive to catecholamines.^78^ The presumably sympathetic symptoms were the motivations for the use of sympathetic blocks to treat CRPS. However, meta-analyses with inconclusive findings raised doubts.^67^ Today, sympathetic blocks should be an exception rather than a rule for CRPS treatment.

The role of the recently discovered agonistic auto-antibodies against adreno- and acetylcholine-receptors in the generation of autonomic symptoms or pain is to be clarified in future studies.^30,50^

### 5.4. Psychosocial factors

Depression and anxiety, which were assessed by self-reports, are not related to the development of CRPS.^7^ However, it would be naive to suppose that only in CRPS, on the contrary to all other chronic pain diseases, psychosocial factors would not be involved particularly in perpetuation of pain, suffering, and reduced participation. In comparison to patients with limb pain (10% in limb pain controls and 4% in healthy subjects), 38% of patients with CRPS report posttraumatic stress symptoms after life events before CRPS.^85^ Patients with CRPS also reported more depersonalization phenomena than limb pain controls on the 29 items Cambridge Depersonalization Scale.^60^ In CRPS with fixed dystonia, a somatoform movement disorder is expected in more than 25%.^81^ Anxiety, pain-related fears, and perceived disability are negative predictors for the treatment success after 1 year.^6^

The research on social factors in CRPS is in its infancy. Surprisingly, it is the affluent patients, who develop CRPS more often after distal radius fracture.^21^ Because CRPS develops mainly after trauma, it is not surprising that many patients are involved in lawsuits or compensation claims.^1,52^ Not specifically for CRPS but for “persisting limb pain,” external attributions of responsibility for the injury, and psychological distress were predictors of significant pain 6 months after an orthopaedic trauma in a prospective study. In addition, poor recovery expectations were predictors of pain-related work disability, and being injured at work a predictor of pain severity.^20^ Furthermore, patients with compensation claims, greater financial worry, and actual or perceived injustice (consulting a lawyer, attributing fault to another, and sustaining compensable injury) led to an increase in the risk of failing to return to work.^35^ High perceived injustice was correlated to low education and prevented return to work 12 months after injury.^43^

## 6. Treatment options

A problem of multi-faceted pathophysiology is that a “one fits all”-treatment for CRPS will probably never be available. Another big problem with CRPS therapy is that high-quality randomized controlled multicentre trials (RCTs) are missing. Most RCTs are single centre or lack an active placebo arm. Furthermore, since the publication of highly recognized negative trials for pain relief through vertebroplasty, which used an adequate control arm,^14,47^ CRPS intervention studies must be interpreted with care. Nevertheless, treatment options, which were supported by an RCT, are marked by “RCT” in this review. However, the section is influenced by the authors' experiences from 25 years of CRPS care. A systematic approach would have to report mainly “no firm evidence.”^68^

Because of the above-mentioned constellations in CRPS pathophysiology, the following basic therapeutic principles evolve:

(1) Medical and nonmedical pain therapy (acute and chronic phases)
(2) Physiotherapy, occupational therapy and training therapy (acute and chronic phases)
(3) Anti-inflammatory therapy (acute phase)
(4) Psycho- and sociotherapy in a multimodal treatment setting (especially targeting pain-related fears; all phases if necessary)
(5) A limited number of sympathetic nerve blocks (in selected cases after successful test blocks, in specialized centres)
(6) Therapy of dystonia (only at specialized centres).

In the following, therapeutic recommendations for CRPS are described referring also to the guidelines published by eg, the German Association for Neurology (www.dgn.org./leitlinien.html).

### 6.1. Medical and nonmedical pain therapy (acute and chronic phases)

There is no firm proof of efficacy in CRPS for drugs used in other chronic neuropathic pain disorders. A very moderate effect on allodynia was shown for gabapentin (RCT; secondary end point).^91^ It is justified to assume that this might be valid also for pregabalin. Sedative tricyclic antidepressants should be used in particular if sleeping problems prevail. Although there are no controlled studies, analgesic drugs according to the World Health Organization analgesic ladder can be tested, especially in very acute phases. If opioids are chosen,^37^ we suggest that a clear efficacy (eg, reduction of pain >> 50% with reasonable doses) must be demonstrated within 2 weeks. The efficacy of opioids must be strictly controlled. Otherwise, opioid-insensitive pain leads to false increase of dosage, habituation, dependence, and finally increase in pain (opioid-induced hyperalgesia^76^). Opioid-insensitive pain might be frequent because of decreased central opioid receptor availability in CRPS.^49^

There might be a reduction in pain for up to 3 months after intravenous ketamine (continuous infusion for 4 days; maximum 30 mg/h for a 70-kg patient). There are 3 RCTs and many case reports. For the exact protocol, the original publication should be studied.^82^ We want to indicate that blinding must have been incomplete because of the obligate psychotropic side effects of ketamine (vs saline). Accordingly, most systematic reviews and meta-analyses came to the conclusion of “low quality evidence” for ketamine.^23^ If treatment courses are repeated, liver and psychiatric side effects must be monitored.

After failure of noninvasive therapies, spinal cord stimulation (SCS) seems to be an alternative to treat CRPS pain but not function in the lower extremity for up to 5 years (RCT, no active control).^48^ SCS for the upper extremity might be problematic because of complications like dislocations of the electrodes. Equally promising seems the stimulation of dorsal root ganglia (DRG). One controlled study (RCT) has been published in which patients were treated either with DRG stimulation or with SCS.^28^ The devices were implanted only after positive test stimulations. The outcome of the DRG stimulation regarding pain reduction and quality of life was superior to SCS. Like in all interventions, the outcome is dependent on the surgeon's expertise. Relevant psychological comorbidities must be excluded beforehand. Otherwise, there will be further traumatization^70^ and reduced efficacy.^16^

### 6.2. Anti-inflammatory therapy (acute phase)

Not only in our hands glucocorticoids reduce posttraumatic inflammation.^19,100^ We have good experiences with the administration of high initial doses of oral glucocorticoid (100 mg prednisolone per day), which is then tapered down by 25 mg every 4 days.^100^ Under the assumption of an ongoing inflammation for 3 to 6 months,^10^ higher doses (eg, 500–1000 mg methylprednisolone intravenously as in multiple sclerosis therapy) and longer treatment courses (as in all autoimmune diseases) would make more sense but high-quality randomized controlled trials for steroids are lacking, and therefore we follow the principle of “primum non nocere.” Pathophysiologically, steroids make sense during the acute phase.

Bisphosphonates are drugs which are best investigated for CRPS; there are positive studies (several mainly mono-centre RCTs) for nearly all bisphosphonates available in the market.^18^ They not only reduce osteoclast activity, but also inhibit posttraumatic inflammation.^95^ Alendronate is administered either orally with a high dose of 40 mg/d across 8 weeks, or intravenously with a dosage of 7.5 mg for 3 consecutive days. Clondronate is administered intravenously with a dosage of 300 mg for 10 consecutive days; pamindronate with a single dosage of 60 mg, and neridronate 4 times with 100 mg every third day. Whether bisphosphonates are a reasonable treatment only for acute or also for chronic CRPS has to be debated. Pathophysiologically, they make more sense for acute CRPS. We want to make a personal comment. In our hands, bisphosphonates seem to be of limited value in particular after steroid treatment.

In the Netherlands, the application of dimethylsulfoxide 50% as a fatty basis cream—3 times daily on the affected extremity—is a standard procedure. Dimethylsulfoxide traps free radicals, which are produced during inflammation and ischemia. Dimethylsulfoxide had a positive impact on a composite score but not specifically on pain (RCT).^73,102^

### 6.3. Physiotherapy/occupational therapy/training therapy (acute and chronic phases)

Physical and occupational therapies accomplish reduction of pathologic movement patterns and movement limitations and train a physiologic use of the extremity. Patients should be encouraged to voluntarily use the affected extremity even if this involves a temporary increase in pain and other symptoms. The safety of such an approach has been demonstrated.^90^ There is still a widespread misconception that patients with CRPS should avoid pain to prevent an aggravation; this is not valid. If the extremity is not moved during the inflammatory phase when a proliferation of connective tissue cells occurs, contractures follow quickly. On the contrary, painful interventions by others, against the will of the patients, eg, passive movements by therapists or less empathic physicians, should be avoided because of a loss of patients' self-control.

Mirror therapy involves learning to adapt the mirror image of the healthy extremity as the affected limb. This should reduce pain and subsequently improve movement. Mirror therapy works best with acute CRPS and CRPS after stroke (small RCT, only for poststroke CRPS)^15^ and is a standard procedure for experienced physiotherapists. An advancement of mirror therapy is “graded motor imagery.” Initially, this includes recognition of right and left extremities on a monitor; a second step is the imagination of movements of the affected extremity, and the third step is the mirror therapy itself. Efficacy was high in single-centre studies (RCT)^62,63^ but not reproduced in an open multi-centre trial, which should usually be even more sensitive to placebo effects.^46^ “Pain Exposure Physical Therapy” (including passive treatments) is performed with the patients' consent, ignoring pain. Pain is not further mentioned throughout the treatment. In an open study, this approach led to an improvement of function and to an improvement of pain. This effect could not be confirmed in an RCT, drop outs were high.^3^ In general, meta-analyses came to the conclusion that there is still a lack of high quality studies for these components of CRPS treatment.^68^

### 6.4. Psycho- and sociotherapy in a multimodal treatment setting (especially targeting pain-related fears; all phases)

Psychotherapeutic and sociotherapeutic methods represent an important part of multimodal pain therapy, especially if accompanying psychosocial factors or comorbidities exist (eg, depressive mood, pain-related avoidance, posttraumatic stress disorders, perceived injustice, and financial worries).^35,83,99^ The authors' experiences are that patients with many psychosocial problems seem to be harder to treat. This is not due to the desire for compensation; the reason could be a deep uncertainty regarding future perspectives. This is not beneficial for an active participation in treatment.^24,29^

“Graded Exposure” (GEXP) treatment has shown good evidence for efficacy in CRPS. For this approach, a psychologist identifies and classifies fear-triggering situations (eg, pain induction through certain movements and situations). Patients are then gradually exposed to these situations by a physiotherapist. The efficacy of graded exposure in comparison to conventional rehabilitative therapy was confirmed in 1 large case series for chronic CRPS (n = 106) and a recently published small and monocentre RCT.^29^ Graded exposure reduced pain and improved function.

### 6.5. A limited number of sympathetic nerve blocks (in selected cases after successful test blocks, in specialized centres)

A recent Cochrane analysis could not reveal evidence for the efficacy of sympathetic blocks^67^ because of the lack of high-quality studies. This means a definite negative assumption is also not possible. In our view and according to consensus of experts, who contributed to the German CRPS treatment guideline, a series of sympathetic blocks under strict control of the therapeutic effect (pain reduction >> 50%) throughout 5 weeks (twice a week) can be tried,^4^ if a test block in the beginning was successful. Such a series should be prematurely stopped if single blocks became unsuccessful, or conversely, if long-lasting therapeutic effects have been achieved. Sympathetic blocks are not first-line therapy and should only be conducted by an experienced pain therapist.

### 6.6. Therapy of dystonia (only at specialized centres)

**Figure 1.**
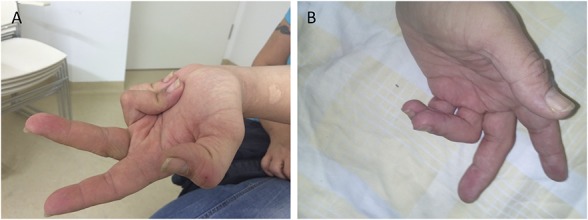
CRPS-related fixed dystonia (A) before and (B) after botulinum toxin A treatment. Pain improves in parallel with a reduction in muscle contraction. CRPS, complex regional pain syndrome.

**Figure 2.**
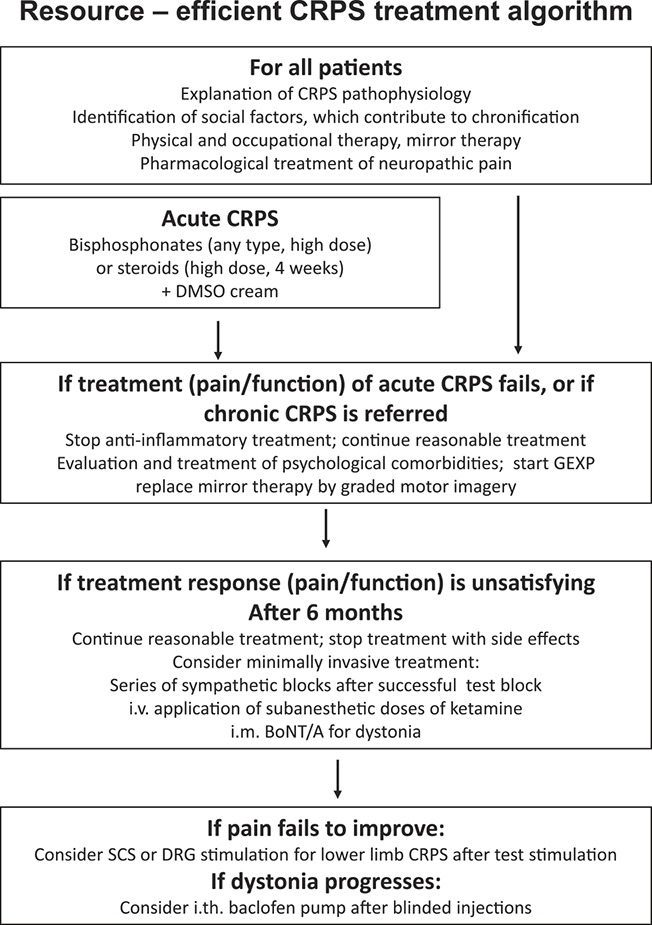
The authors' suggestion of a resource-effective treatment algorithm for CRPS (details see text). Under the assumption that meta-analyses request “better or more RCTs” for any CRPS therapy, we suggest a stepwise approach starting with noninvasive treatment with proportionate risks (“nihil nocere”). Nonpharmacological treatment is intensive but not too time consuming at the beginning. Evaluation of psychological factors, which prevent improvement, follows early, and nonpharmacological treatment becomes more intense. Invasive treatment should be performed only by specialists; minimally invasive procedures should be tried before implantation of neuroprosthetics. BoNT/A, botulinum toxin type A; CRPS, complex regional pain syndrome; DMSO, dimethylsulfoxide; DRG, dorsal root ganglion, (stimulation); GEXP, graded exposure in vivo; i.m., intramuscular; i.v., intravenous; SCS, spinal cord stimulation.

Botulinum toxin might be less effective for treatment of fixed dystonic posturing in CRPS than for action-related dystonia in neurology.^79^ However, we agree that because of the minimally invasive character, a therapeutic attempt makes sense in selected cases (Fig. 1, clinical diagnostic criteria for CRPS are fulfilled). Our experiences show that dosage and number of treated muscles must be high enough to reduce muscle strength. If botulinum toxin improves dystonia, pain also improves. In case studies, successful treatment of dystonia has been shown during continuous intrathecal application of baclofen by a pump.^92^ This treatment, however, must be performed by experienced centres and only after vigorous assessment^32^ including psychological factors.^40^ Complications like catheter dislocation or break and cerebrospinal fluid leakage with postural headaches are frequent. Firm evidence that both treatments for CRPS dystonia are successful is sparse (Fig. 2).

## 7. Outlook

Complex regional pain syndrome is a “visible” pain disease. During the past years, there has been significant progress in understanding the pathophysiology, which will ultimately lead to better individualized treatment. It is important to explain the pathophysiology of CRPS as good as we know and the purpose of each therapy to the patients, and to motivate them to actively participate by developing self-management strategies. Thereby, therapeutic success might become better. Whether the treatment success is sufficient to reintegrate patients back into their previous lives also depends on nonmedical factors. In any case, we urgently need multicentre RCTs, which have to be performed by closely cooperating networks.

## Disclosures

The authors have no conflict of interest to declare.

The authors were supported by intramural funds of the University Medical Center Mainz to V. Dimova and the Berufsgenossenschaft für das Gesundheitswesen Mainz and the EU, FP7 under grant agreement number 602133 to F. Birklein.
